# Resveratrol Inhibition of the WNT/β-Catenin Pathway following Discogenic Low Back Pain

**DOI:** 10.3390/ijms23084092

**Published:** 2022-04-07

**Authors:** Tiziana Genovese, Daniela Impellizzeri, Ramona D’Amico, Marika Cordaro, Alessio Filippo Peritore, Rosalia Crupi, Enrico Gugliandolo, Salvatore Cuzzocrea, Roberta Fusco, Rosalba Siracusa, Rosanna Di Paola

**Affiliations:** 1 Department of Chemical, Biological, Pharmaceutical and Environmental Sciences, University of Messina, Viale Ferdinando Stagno D’Alcontres 31, 98166 Messina, Italy; tgenovese@unime.it (T.G.); dimpellizzeri@unime.it (D.I.); rdamico@unime.it (R.D.); aperitore@unime.it (A.F.P.); salvator@unime.it (S.C.); rsiracusa@unime.it (R.S.); 2 Department of Biomedical, Dental and Morphological and Functional Imaging, University of Messina, Via Consolare Valeria, 98125 Messina, Italy; marika.cordaro@unime.it; 3 Department of Veterinary Sciences, University of Messina, 98168 Messina, Italy; rcrupi@unime.it (R.C.); egugliandolo@unime.it (E.G.); dipaolar@unime.it (R.D.P.); 4 Department of Pharmacological and Physiological Science, Saint Louis University School of Medicine, Saint Louis, MO 63104, USA; 5 Department of Clinical and Experimental Medicine, University of Messina, Via Consolare Valeria, 98125 Messina, Italy

**Keywords:** biochemistry, resveratrol, pain

## Abstract

Low back pain (LBP) management is an important clinical issue. Inadequate LBP control has consequences on the mental and physical health of patients. Thus, acquiring new information on LBP mechanism would increase the available therapeutic tools. Resveratrol is a natural compound with many beneficial effects. In this study, we investigated the role of resveratrol on behavioral changes, inflammation and oxidative stress induced by LBP. Ten microliters of Complete Freund’s adjuvant (CFA) was injected in the lumbar intervertebral disk of Sprague Dawley rats to induce degeneration, and resveratrol was administered daily. Behavioral analyses were performed on day zero, three, five and seven, and the animals were sacrificed to evaluate the molecular pathways involved. Resveratrol administration alleviated hyperalgesia, motor disfunction and allodynia. Resveratrol administration significantly reduced the loss of notochordal cells and degenerative changes in the intervertebral disk. From the molecular point of view, resveratrol reduced the 5th/6th lumbar (L5–6) spinal activation of the WNT pathway, reducing the expression of WNT3a and cysteine-rich domain frizzled (FZ)8 and the accumulation of cytosolic and nuclear β-catenin. Moreover, resveratrol reduced the levels of TNF-α and IL-18 that are target genes strictly downstream of the WNT/β-catenin pathway. It also showed important anti-inflammatory activities by reducing the activation of the NFkB pathway, the expression of iNOS and COX-2, and the levels of PGE2 in the lumbar spinal cord. Moreover, resveratrol reduced the oxidative stress associated with inflammation and pain, as shown by the observed reduced lipid peroxidation and increased GSH, SOD, and CAT activities. Therefore, resveratrol administration controlled the WNT/β-catenin pathway and the related inflammatory and oxidative alterations, thus alleviating the behavioral changes induced by LBP.

## 1. Introduction

Low back pain (LBP) is a very common clinical symptom that occurs during middle and old age [1]. It affects over 40% of people during lifetime [2]. From 1990 to 2015, LPB caused an increase in the number of adults with disabilities of 54% [3]. LPB has also consequences on the mental and physical health of patients, placing a burden on the social support system and health care [4]. It is often caused by lumbar intervertebral disk and spinal disease, causing symptoms like neuropathic pain and motor dysfunction [5]. The pathologic degeneration of intervertebral disks is characterized by the loss of biological, biomechanical, and architectural properties and induces chronic spinal inflammation and LPB. Although many treatments have been employed for discogenic pain management, their clinical efficacy remains debatable. Analgesics, in particular opioids, are the primary treatment method for chronic pain [6]. Unfortunately, their abuse and misuse are increasingly prevalent, and their side effects contribute to the decline of the health and quality of life of patients [7]. Current treatment options for discogenic back pain range from medicinal anti-inflammation strategies to invasive procedures including spine fusion and, recently, spinal arthroplasty. However, these treatments are limited to relieving symptoms, with no attempt to restore the disc’s structure. Recently, there has been a growing interest in developing strategies that aim to repair or regenerate the degenerated disc biologically. Indeed, the pathophysiologic mechanism underlying the nociceptive degeneration of lumbar intervertebral disks needs to be fully defined. Thus, investigating the role of spinal activation in LBP is an important challenge. It has been shown that the neuronal modifications that occur in neuropathic, bone cancer and post-operative pain require the activation of the WNT/β-catenin signaling [8]. Wnts are a family of proteins that modulate cellular processes such as proliferation, differentiation and migration [9,10]. This family includes 19 members of secreted lipid-modified signaling mediators [11]. In the absence of WNT ligands, β-catenin is located in the cytoplasm bound to a destruction complex consisting of the scaffolding proteins Axin and APC and two kinases, i.e., glycogen synthase kinase 3β (GSK3β) and casein kinase 1 (CK1). This complex traps and phosphorylates β-catenin, thereby targeting it for ubiquitinoylation and proteasomal degradation. When WNT ligands binds the cysteine-rich domain frizzled (FZ) and LRP5/6 receptors on the cell membrane, the destruction complex is destroyed, and β-catenin is not phosphorylated and degraded. After accumulating into the cytoplasm, β-catenin is able to translocate into the nucleus and induce the transcription of target genes such as IL-18 and TNF-α [12,13]. Many studies, in fact, have shown that the inflammatory response activated by WNT/β-catenin signaling has a key role in the development of low back pain [14,15]. Cytokines overexpression within the dorsal horn of the spinal cord is associated with pain behaviors [16,17,18]. These cytokines have a key role in the development of neuropathic pain by activating other transcription factors that contribute to the chronic hypersensitivity and persistence of pain perception [19,20,21]. This inflammatory condition also induces the overexpression of reactive oxygen species (ROS), hydrogen peroxide (H_2_O_2_), nitric oxide (NO) and superoxide, which are involved in the sensitization process [22].

Thus, promising compounds to treat LBP would be molecules that target the WNT/β-catenin pathway, with anti-inflammatory and antioxidant properties. Resveratrol is a largely used inhibitor of the WNT pathway. It is a natural polyphenolic substance that can be isolated from plants. Resveratrol has many physiological effects including cardiovascular and anticancer [23,24,25,26] effects. It downregulates TCF4, a key regulator of WNT transcription, through the stimulation of the proteasome complex [27] and showed its effect in many tissues and models. In particular, resveratrol showed inhibitory effects in RKO and HT29 colon cancer cells [28], glioma cells [29,30], MGC-803 and Colo16 cells [31], U2-OS cells [32] and cardiomyocytes [33]. Resveratrol showed anti-inflammatory effects in a rat model of arthritis by inhibiting WNT activity [34]. It also has well-described antioxidant properties [35,36,37]. The aim of this study was to evaluate the effect of resveratrol on the perception of LBP, through the modulation of the WNT/β-catenin pathway, inflammation and oxidative stress.

## 2. Results

### 2.1. Effect of Resveratrol Administration on Intravertebral Disc Degeneration

**Figure 1:**
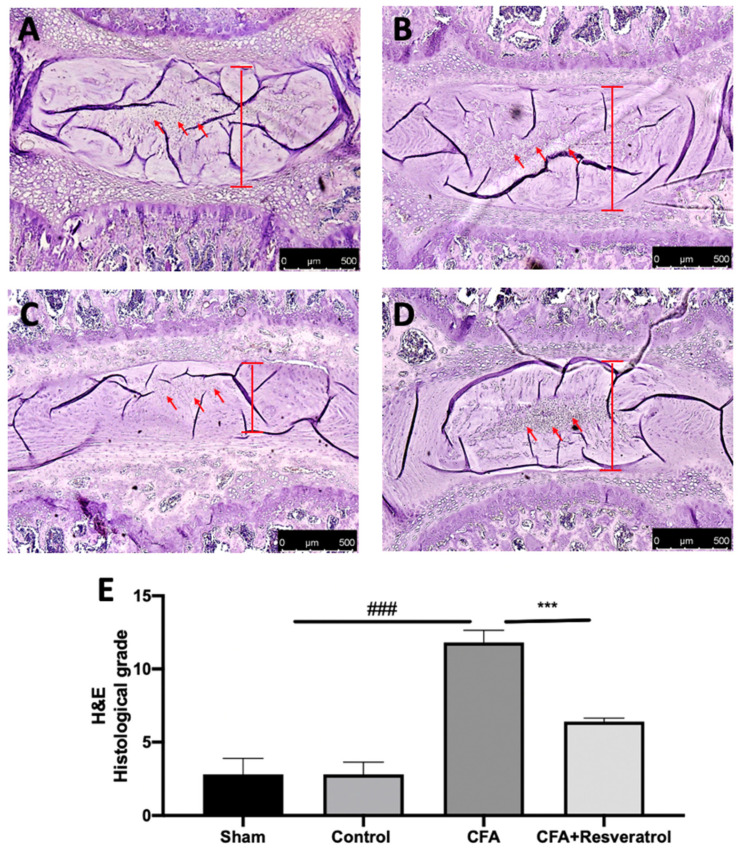
Resveratrol administration reduced intravertebral disk degeneration. Hematoxylin and Eosin staining: Sham (**A**), Control (**B**), CFA (**C**), CFA + Resveratrol (**D**), Histological score (evaluation of anulus fibrosus and nucleus pulposus) (**E**). ### *p* < 0.001 vs. sham, *** *p* < 0.001 vs. CFA. Error bar shown as SEM.

This study investigated the effect of resveratrol administration in an animal model of LBP. Ten microliters of Complete Freund’s adjuvant (CFA) was injected in the lumbar intervertebral disk of Sprague Dawley rats to induce degeneration, and resveratrol was administered daily by gavage until the seventh day. On the seventh day, behavioral analyses were performed, and the animals were sacrificed. Histological findings regarding the intervertebral disc volume showed that the nucleus pulposus contained abundant notochordal cells surrounded by large zones of extracellular matrix, and the cartilaginous endplates were hyaline cartilages composed of chondrocytes in the sham group (Figure 1A,E). In contrast, in the CFA-injected group, the disc showed degenerative changes and reduced notochordal cells in the nucleus pulposus, increased disorganized hypocellular fibrocartilaginous tissue and lower disc height (Figure 1C,E). Resveratrol administration significantly reduced these degenerative changes (Figure 1D,E). No changes were detected in animals injected with saline (Control group) (Figure 1B,E) compared to the sham group. The results were examined by one-way ANOVA followed by a Dunnett’ post-hoc test for multiple comparisons (F(3,16), *p* < 0.0001).

### 2.2. Effect of Resveratrol Administration on Notochordal Cells and Cartilage Degeneration

**Figure 2:**
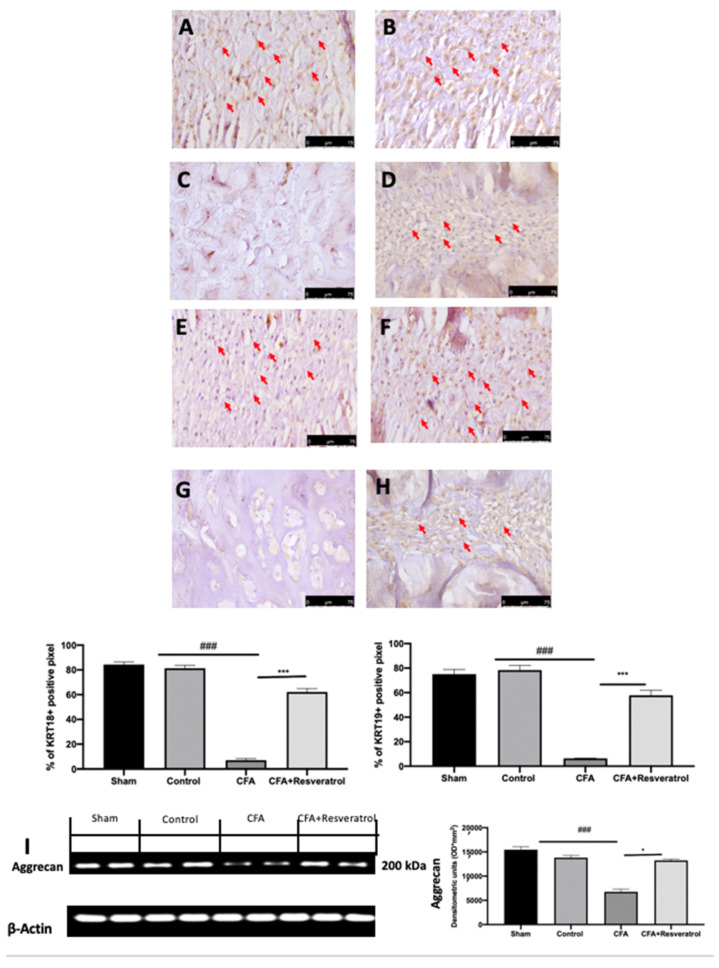
Resveratrol administration restored notochordal cells and cartilage markers. Immunohistochemical analysis of KRT18: Sham (**A**), Control (**B**), CFA (**C**), CFA + Resveratrol (**D**); Immunohistochemical analysis of KRT19: Sham (**E**), Control (**F**), CFA (**G**), CFA + Resveratrol (**H**); Western Blot analysis of Aggrecan (**I**). * *p* < 0.05 vs. CFA, ### *p* < 0.001 vs. sham, *** *p* < 0.001 vs. CFA. Error bar shown as SEM.

Immunohistochemical analysis of keratin (KRT) 18 and KRT19, notochord-specific markers, was conducted to evaluate the notochordal cells in the nucleus pulposus. Positive KRT18 and KRT19 staining was detected in samples collected from the sham group (Figure 2A,E), whereas no changes were detected in animals injected with saline (Control group) (Figure 2B,F) as compared to the sham group. Both notochord-specific markers were significantly decreased in the CFA group (Figure 2B,G), while resveratrol administration after CFA injection partially restored notochordal cells and markers staining (Figure 2D,H). Western Blot analysis of cartilage tissue also showed a reduced expression of Aggrecan, one of the major structural components of the extracellular matrix, in the CFA group, while its levels were increased in animals treated with resveratrol (Figure 2I). The results were examined by one-way ANOVA followed by Dunnett post-hoc test for multiple comparisons (KR18 (F(3,16) *p* < 0.0001); KR19 (F(3,16) *p* < 0.0001); Aggrecan (F(3,4) *p* < 0.0001)).

### 2.3. Effect of Resveratrol Administration on Osteochondral Remodeling of the Endplate

**Figure 3:**
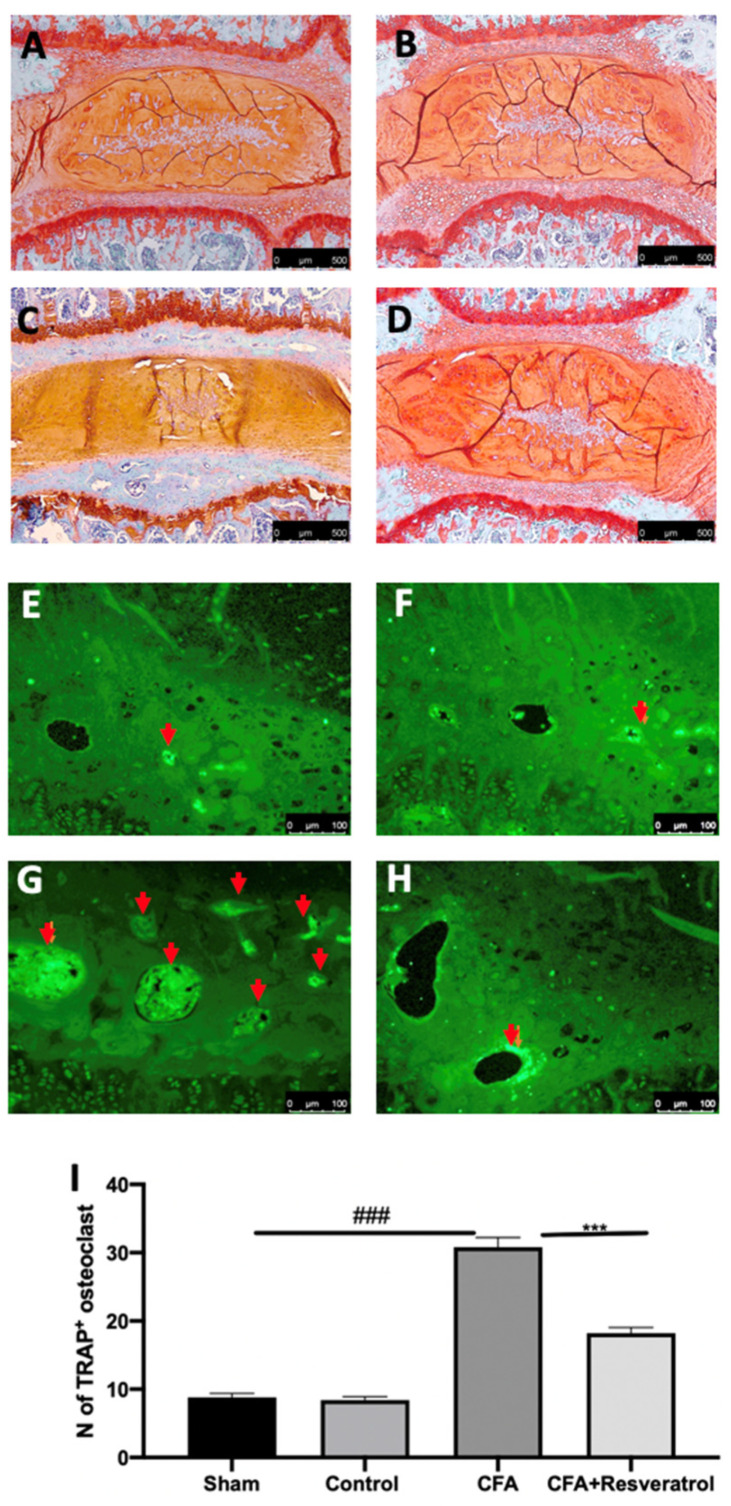
Resveratrol administration reduced osteoclast activation. Safranin-O and fast green staining: Sham (**A**), Control (**B**), CFA (**C**), CFA + Resveratrol (**D**); Immunofluorescence analysis of TRAP: Sham (**E**), Control (**F**), CFA (**G**), CFA + Resveratrol (**H**), N of TRAP+ osteoclast (pointed by the arrows) (**I**). ### *p* < 0.001 vs. sham, *** *p* < 0.001 vs. CFA. Error bar shown as SEM.

Safranin O and fast green staining, used to assess proteoglycan loss, suggested that the endplates underwent endochondral ossification after CFA injection (Figure 3C), as compared to the sham group (Figure 3A). No changes were detected in animals injected with saline (Control group) (Figure 3B) as compared to the sham group (Figure 3A). Resveratrol administration reduced the damage of the endplate structure and the calcification and ossification mechanisms (Figure 3D). Immunofluorescence analysis of TRAP indicated bone turnover of the intervertebral disc volume. CFA injection significantly increased osteoclast activation in the ossification region of the endplate (Figure 3G,I), as compared to the sham group (Figure 3E,I). Resveratrol administration also reduced the resorption cavities in the secondary ossification center above the endplates, as shown by the reduced number of TRAP^+^ osteoclasts (Figure 3H,I). No changes were detected in animals injected with saline (Control group) (Figure 3F,I) as compared to the sham group (Figure 3E); thus, further analyses were performed on the sham group. The results were examined by one-way ANOVA followed by Dunnett post-hoc test for multiple comparisons (TRAP (F(3,16) *p* < 0.0001)).

### 2.4. Effect of Resveratrol Administration on Mechanical Allodynia, Motor Dysfunction and Thermal Hyperalgesia

**Figure 4:**
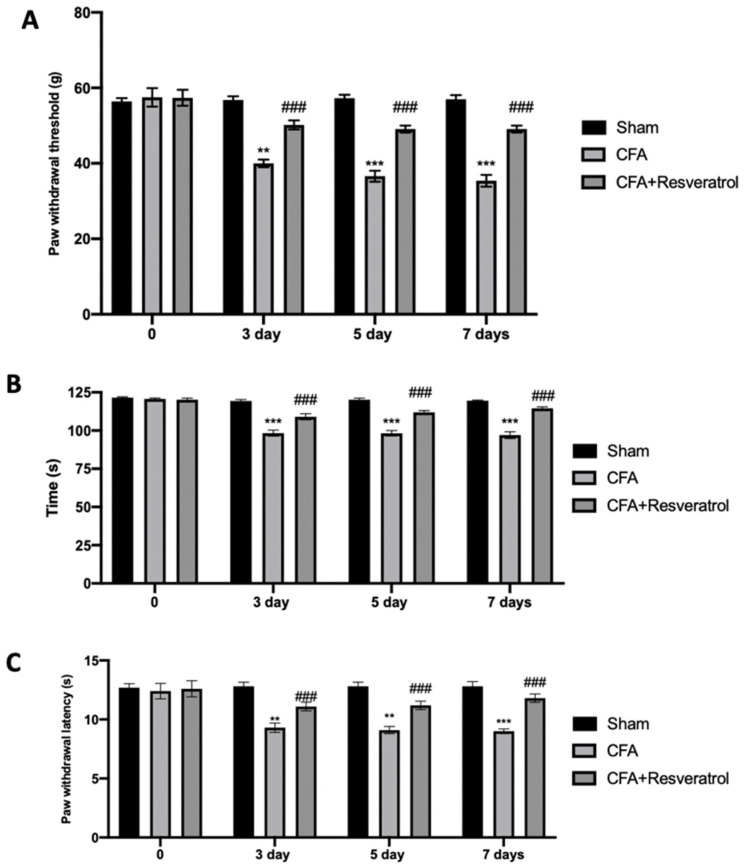
Resveratrol administration reduced mechanical allodynia, motor dysfunction and thermal hyperalgesia: Von Frey Test (**A**), Rotarod Test (**B**) and Plantar Test (**C**). ** *p* < 0.01 vs. CFA, ### *p* < 0.001 vs. sham, *** *p* < 0.001 vs. CFA. Error bar shown as SEM.

Behavioral probes were used to investigate resveratrol effect on LBP perception. CFA-injected rats showed increased mechanical allodynia, which was reduced in animals treated with resveratrol after CFA injection (Figure 4A). The rotarod test showed motor impairments in CFA-injected animals, while reduced locomotor impairments were detected in resveratrol-administered rats (Figure 4B). The plantar test displayed increased thermal hyperalgesia, which was reduced in animals treated with resveratrol after CFA injection (Figure 4C). The results were examined by one-way ANOVA followed by Dunnett post-hoc test for multiple comparisons (Mechanical allodynia (F(6108) *p* < 0.0001); Motor impairments (F(6108) *p* < 0.0001); Thermal hyperalgesia (F(6108) *p* = 0.0005)).

### 2.5. Effect of Resveratrol Administration on Pain-Related Signaling

**Figure 5:**
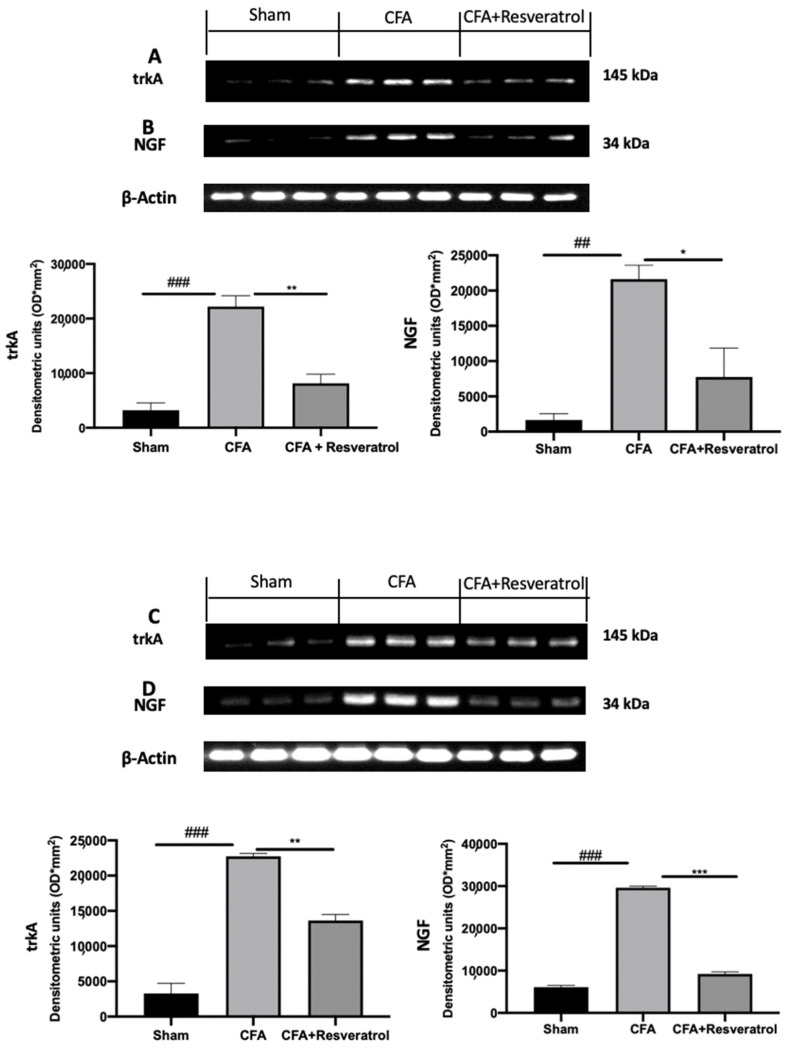
Resveratrol administration reduced pain-related signaling. Western blot analysis of: trkA (**A**), NGF (**B**) expression in the disc tissue; Western blot analysis of: trkA (**C**), NGF (**D**) expression in the spinal cord tissue. * *p* < 0.05 vs. CFA, ## *p* < 0.01 vs. sham, ** *p* < 0.01 vs. CFA, ### *p* < 0.001 vs. sham, *** *p* < 0.001 vs. CFA. Error bar shown as SEM.

Western blot analyses were conducted to evaluate pain mediators’ expression. Increased trkA (Figure 5A) and NGF (Figure 5B) expression was detected in disc tissue collected from CFA-injected rats as compared with sham samples. Additionally, spinal cord samples harvested from CFA-injected rats showed upregulated expression of both pain mediators (Figure 5C,D). Resveratrol administration reduced trkA and NGF expression in both tissues. The results were examined by one-way ANOVA followed by Dunnett post-hoc test for multiple comparisons (Disk tissue: trkA (F(2,6) *p* = 0.0005); NGF (F(2,6) *p* = 0.0050); Spinal cord tissue: trkA (F(2,6) *p* < 0.0001); NGF (F(2,6) *p* < 0.0001)).

### 2.6. Effect of Resveratrol Administration on WNT/β-Catenin Pathway

**Figure 6:**
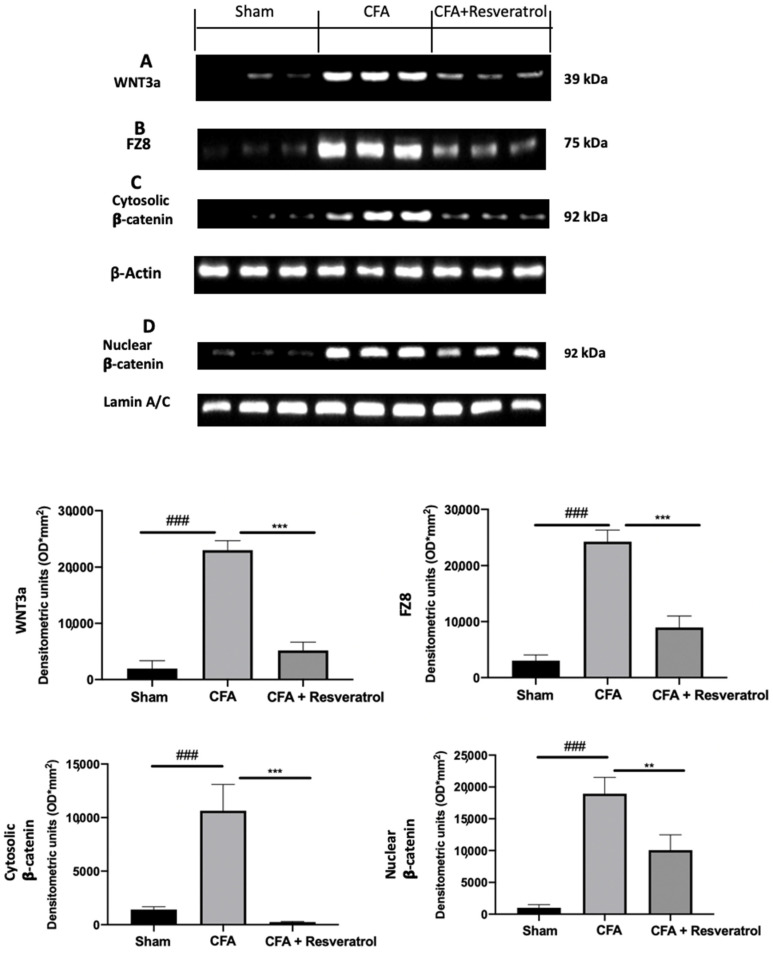
Resveratrol administration reduced WNT/β-catenin pathway activation. Western blot analysis of: WNT3a (**A**), FZ8 (**B**), cytosolic β-catenin (**C**) and nuclear β-catenin (**D**) expression. ** *p* < 0.01 vs. CFA, ### *p* < 0.001 vs. sham, *** *p* < 0.001 vs. CFA. Error bar shown as SEM.

Increased WNT3a (Figure 6A) and FZ8 (Figure 6B) expression was detected in samples collected from CFA-injected rats as compared with sham samples. Additionally, CFA-injected animals showed increased β-catenin accumulation in both cytosolic (Figure 6C) and nuclear (Figure 6D) compartments. Samples harvested from resveratrol-treated rats showed reduced WNT3a (Figure 6A) and FZ8 (Figure 6B) expression, as well as reduced β-catenin accumulation in both cellular compartments (Figure 6C,D). The results were examined by one-way ANOVA followed by Dunnett post-hoc test for multiple comparisons (WNT3a (F(2,6) *p* < 0.0001); FZ8 (F(2,6) *p* < 0.0001); cytosolic β-catenin (F(2,6) *p* = 0.0002); nuclear β-catenin (F(2,6) *p* = 0.0001)).

### 2.7. Effect of Resveratrol Administration on Cytokine Expression and the NFkB Pathway

**Figure 7:**
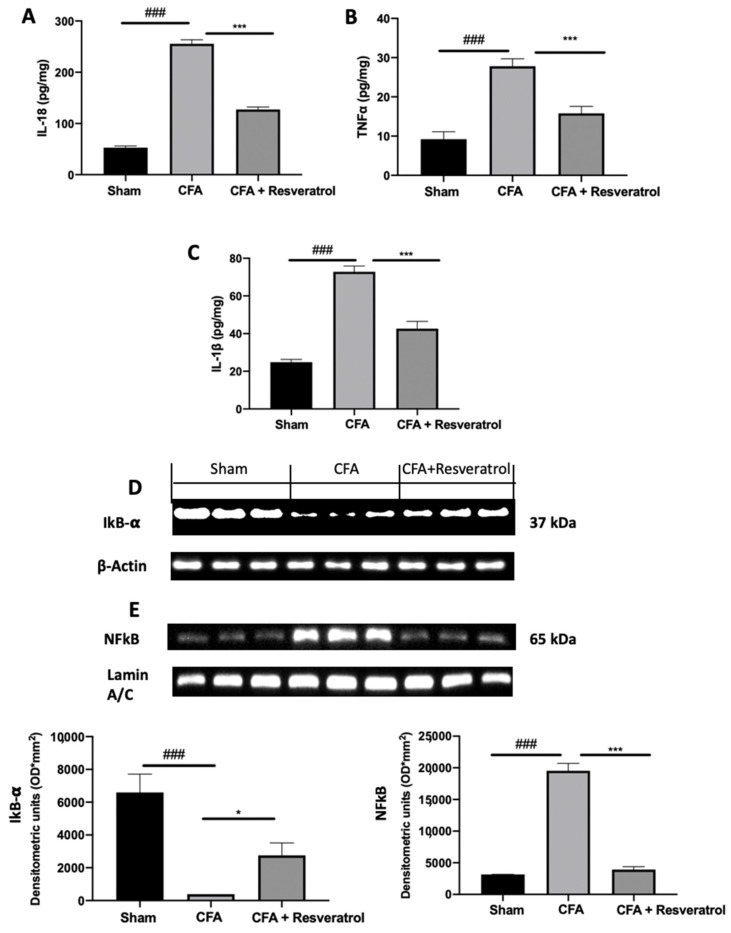
Resveratrol administration decreased cytokines expression and NFkB pathway activation. IL-18 (**A**), TNF-α (**B**) and IL-1 β (**C**), Western blot analysis of: IkB-α (**D**) and NFkB (**E**) expression. * *p* < 0.05 vs. CFA, ### *p* < 0.001 vs. sham, *** *p* < 0.001 vs. CFA. Error bar shown as SEM.

In order to evaluate the activation of inflammatory pathways, cytokines expression was assessed. Upregulated levels of IL-18 (Figure 7A), TNF-α (Figure 7B) and IL-1β (Figure 7C) were found in samples from the CFA-treated group as compared to those from sham-treated animals. Resveratrol treatment significantly reduced their levels (Figure 7A–C). Resveratrol administration importantly increased cytosolic IkB-α expression, which was reduced by CFA injection, restoring it to the sham levels (Figure 7D). Western blot analysis also showed reduced nuclear NFkB expression in samples harvested from resveratrol-administered rats (Figure 7E), as compared to those form CFA-injected animals. The results were examined by one-way ANOVA followed by Dunnett post-hoc test for multiple comparisons (IL-18 (F(2,12) *p* < 0.0001); TNF-α (F(2,12) *p* < 0.0001); IL-1β (F(2,12) *p* < 0.0001); IkB-α (F(2,6) *p* = 0.0002); NFkB) (F(2,6) *p* < 0.0001)).

### 2.8. Effect of Resveratrol Administration on Inflammation

**Figure 8:**
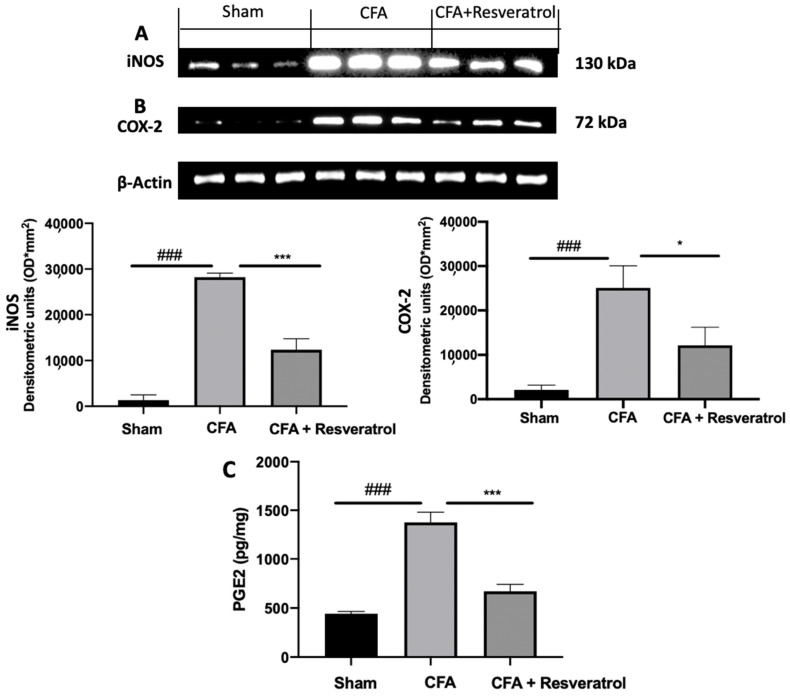
Resveratrol administration reduced iNOS, COX-2 and PGE2 expression. Western blot analysis of: iNOS (**A**), COX-2 (**B**) expression, PGE2 levels (**C**). * *p* < 0.05 vs. CFA, ### *p* < 0.001 vs. sham, *** *p* < 0.001 vs. CFA. Error bar shown as SEM.

Increased expression of iNOS and COX-2 was detected in samples harvested from CFA-injected animals, as compared to samples from sham rats. Resveratrol treatment reduced the levels of both molecules (Figure 8A,B). Resveratrol reduced PGE2 levels as well, as compared to the levels measured in CFA-injected rats (Figure 8C). The results were examined by one-way ANOVA followed by Dunnett post-hoc test for multiple comparisons (iNOS (F(2,6) *p* < 0.0001); COX-2 (F(2,6) *p* = 0.0009); PGE2 (F(2,12) *p* < 0.0001)).

### 2.9. Effect of Resveratrol Administration on Oxidative Stress

**Figure 9:**
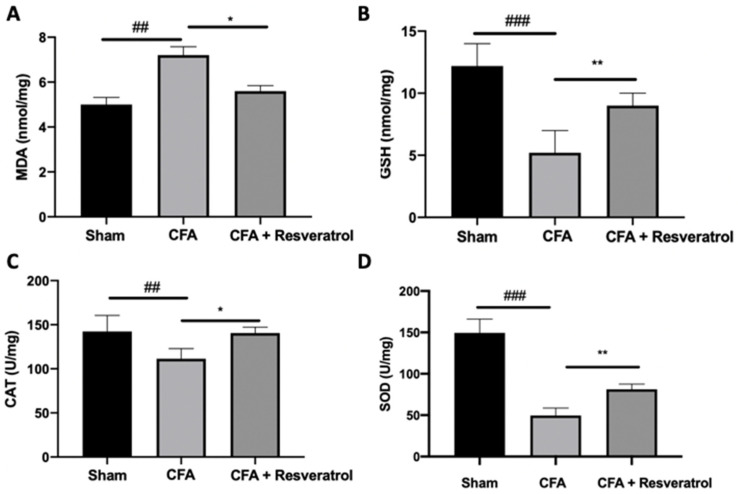
Resveratrol administration reduced oxidative stress. MDA (**A**), GSH (**B**), SOD (**C**), CAT (**D**). * *p* < 0.05 vs. CFA, ## *p* < 0.01 vs. sham, ** *p* < 0.01 vs. CFA, ### *p* < 0.001 vs. sham. Error bar showed as SEM.

Biochemical parameters were investigated to assess the presence of oxidative stress. Upregulated lipid peroxidation was detected in CFA-injected rats, while resveratrol administration reduced MDA levels (Figure 9A). GSH levels, SOD and CAT activity decreased in the CFA group, as compared to the sham animals. Resveratrol increased GSH level (Figure 9B) and SOD (Figure 9C) and CAT (Figure 9D) activities. The results were examined by one-way ANOVA followed by Dunnett post-hoc test for multiple comparisons (MDA (F(2,12) *p* = 0.001); GSH (F(2,12) *p* < 0.0001); CAT (F(2,12) *p* = 0.0043); SOD (F(2,12) *p* < 0.0001)).

## 3. Discussion

This study aimed to evaluate the mechanism of resveratrol effect on LBP in rats. CFA injection into the intervertebral disc induced disc degenerative changes, causing pain-related behaviors and increased the expression of local inflammatory mediators. CFA is a water-in-oil emulsion enclosing mycobacterial cell wall components or heat-killed mycobacteria that induce intense local inflammation at the injection site. Our findings showed histologic degeneration of the intravertebral disks, with reduced notochordal cells in the nucleus pulposus, increased disorganized hypocellular fibrocartilaginous tissue and lower disc height. Additionally, resorption cavities in the secondary ossification center above the endplates were detected. Resveratrol administration significantly reduced intervertebral disc degeneration induced by CFA injection. Well in line with these data, resveratrol displayed appreciably effects on behavioral changes. LBP, in fact, is characterized by nociceptive hypersensitivity and motor impairments. Resveratrol significantly reduced pain perception and locomotor deficiencies, downregulating the molecular mediators of pain perception in both disc and spinal cord. This antinociceptive effect would be attributed to several molecular events activated by resveratrol. Thus, we investigated the molecular mechanism induced by resveratrol administration in a rat model of LBP.

In particular, we explored WNT/β-catenin signaling pathway activation in the spinal cord. This pathway is involved in several development processes [9,38]. It was described to be activated in the spinal cord dorsal horn in many neuropathic pain models, such as those based on chronic constriction injury and sciatic nerve ligation [8,39]. Several studies displayed increased levels of multiple inflammatory mediators in patients, including TNF-α and IL-18 [40,41], whose levels correlated with pain intensity [42]. These two factors are target genes strictly downstream of the WNT/β-catenin pathway [8]. Our molecular analysis showed increased WNT3a expression in the lumbar spinal cord. WNT3a binds the FZ receptors, inducing the activation of the pathway and β-catenin accumulation. Resveratrol reduced WNT3a and FZ8 expression and β-catenin accumulation in the cytosolic and nuclear compartments. Additionally, resveratrol showed important anti-inflammatory activities by reducing IL-18, TNF-α and IL-1β levels and the activation of the NFkB pathway. Resveratrol reduced IkB-α cytosolic degradation and NFkB nuclear expression, downregulating the inflammation associated with pain.

Inflammation is a widely hypothesized mechanism for pain. It is present in many painful conditions [43,44,45,46,47,48,49,50], and its reduction is related to a decrease in pain [51,52,53,54]. The NFkB pathway manages the activation of many proinflammatory factors such as iNOS and COX-2 [55]. COX-2, in turn, is responsible for PGE2 increased levels, which causes nociception after peripheral inflammatory stimuli. Our data confirmed the marked increase in iNOS and PGE2 after CFA injection. Resveratrol administration significantly reduced iNOS and COX-2 expression and PGE2 levels, which are important mediators that sensitize neurons and maintain hyperalgesia. Published studies showed that prostaglandin and NO signaling regulate inflammation and oxidative stress, resulting in hyperalgesia development [56]. It is well known that oxidative stress contributes to inflammation; therefore, both processes may underlie a painful condition [57,58]. Intervertebral disk degeneration induced by CFA injection induces the release of local inflammatory and oxidative mediators. In particular, proinflammatory cytokines overexpression significantly stimulates ROS production [59]. This increased oxidative stress contributes to pain by increasing pathological responses such as neuropathy and inflammation [60]. Several works showed increased lipid peroxidation and decreased CAT and SOD levels and GSH activity in both experimental models and patients [60]. These enzymes assist cells in fixing cellular membranes injured by ROS. In particular, SOD converts the excess of superoxide anions into H_2_O_2_, which is then removed by GSH and CAT [61,62]. Resveratrol administration reduced lipid peroxidation and increased GSH, CAT and SOD activities, helping the antioxidant defense system to neutralize the excess of free radicals associated with pain.

In summary, our findings displayed the impact of resveratrol on the progression and development of LBP. In particular, Wnt signaling, among other pathways in the spinal cord, was associated with the reduction of pain-related behavioral features of rats treated with resveratrol by modulating TNF-α and IL-18 levels and the related spinal inflammation and oxidative stress.

## 4. Materials and Methods

### 4.1. Animals

Eight–nine-week-old male Sprague Dawley rats (200–250 g Envigo, Milan, Italy) were employed. This study was approved by the University of Messina Review Board for the care of animals and followed the Italian regulations (D.Lgs 2014/26 and EU Directive 2010/63).

### 4.2. Surgical Procedures

CFA injection was performed as previously described [63]. The lumbar intervertebral disk L5–6 was punctured with a 26-gauge needle, and 10 μL of CFA was injected.

### 4.3. Experimental Groups

The rats were randomly divided in groups.

Sham group: The rats were anesthetized, but no injection was performed. Distilled water (used to dissolve resveratrol) was administered one hour after anesthesia and daily by gavage until the seventh day.

Control group: The rats were anesthetized, and 10 μL of saline (used to dissolve CFA) was injected for 10 minutes into the disk, using an infusion pump. We employed a 5-0 nylon suture to close the skin. Distilled water (used to dissolve resveratrol ) was administered 1 hour after anesthesia and daily by gavage until the seventh day.

CFA group: The rats were injected with 10 μL of CFA for 10 minutes, using an infusion pump into the disk. We employed a 5-0 nylon suture to close the skin. Distilled water (used to dissolve resveratrol) was administered 1 hour after surgery and daily by gavage until the seventh day.

CFA + Resveratrol group: The rats were injected with 10 μL of CFA for 10 minutes using an infusion pump into the disk. We employed a 5-0 nylon suture to close the skin. Resveratrol (3 mg/Kg) was administered 1 hour after anesthesia and daily by gavage until the seventh day.

The resveratrol dose was chosen based on the literature [64]. Behavioral analyses were performed on day 0, 3, 5 and 7, and the animals were sacrificed 7 days after surgery [14]. Lumbar spinal cords were harvested for molecular analyses.

### 4.4. Behavioral Analysis

For each group, *n* = 5 animals were analyzed, and for each test, three different trials were conducted.

#### 4.4.1. Mechanical Hyperalgesia

The Von Frey filament test evaluated animals’ hypersensitivity to a mechanic stimuli. The apparatus comprises a plastic box placed on a metal mesh floor and a force transducer equipped with a plastic tip [65,66,67]. The withdrawal threshold was defined as the force, in grams, at which the animal withdrew its paw.

#### 4.4.2. Thermal Hyperalgesia

The plantar test was employed to evaluate thermal hyperalgesia. Paw withdrawal latencies were measured with a cutoff time of 15 s to prevent tissue damage [68,69]. The results are expressed as paw withdrawal latency(s).

#### 4.4.3. Motor Coordination

The rotarod test was employed to evaluate motor coordination. The test was performed starting from a constant speed of 25 rpm, then the rotation was increased linearly by 20 rpm. The latency for the first fall over a 4 min period was recorded [70].

### 4.5. Histological Analysis

The areas between the lumbar vertebrae (L5–6) were harvested, fixed and decalcified [71]. In particular, the tissues were fixed for 24 h in a formaldehyde solution (10% in PBS) at room temperature and decalcified in Osteosoft solution (Merck Millipore, Milan, Italy). Next, the samples were dehydrated through a graded series of ethanol and xylene and embedded in BioPlast Plus (Bio Optica, Milan, Italy). Sections (5 μm in thickness) were stained with hematoxylin and eosin or Safranin-O and fast green staining, and the histological score was determined [72].

### 4.6. Immunohistochemical Analysis

Spinal cord and lumbar disc 5–6 tissues were harvested, fixed and decalcified [71]. In [73] particular, the tissues were fixed for 24 h in a formaldehyde solution (10% in PBS) at room temperature and decalcified in Osteosoft solution (Merck Millipore). Three non-sequential sections were chosen from each sample for examination. Next, the samples were dehydrated through a graded series of ethanol and xylene and embedded in BioPlast Plus (Bio Optica, Milan, Italy). Sections (5 μm in thickness) were prepared from the tissues [74,75]. The sections were then incubated overnight with KRT18 (CSB-MA000256, Cusabio Technology, Houston, TX, USA) or KRT19 (CSB-MA000207, Cusabio Technology). The samples were washed with PBS and incubated with secondary antibodies. Specific labeling was identified with a biotin-conjugated goat anti-rabbit IgG and avidin–biotin peroxidase complex (Vector Laboratories, Burlingame, CA, USA) [76]. The stained sections were observed using a Leica DM6 microscope (Leica Microsystems S.p.A., Milan, Italy), following a typical procedure [77].

### 4.7. Immunofluorescence Analysis

Sections (5 μm in thickness) were prepared from the tissues [78]. The sections were incubated with mouse monoclonal anti-TRAP antibody (sc-376875, Santa Cruz Biotechnology, Heidelberg, Germany) in a humidified chamber O/N at 37 °C. The sections were washed with PBS and incubated with an FITC-conjugated anti-mouse Alexa Fluor-488 antibody (Molecular Probes, UK) for 1 h at 37 °C. The sections were observed and photographed using a Leica DM6 microscope (Leica Microsystems S.p.A., Milan, Italy).

### 4.8. Western Blot Analysis

Spinal cord and lumbar disc 5–6 tissues were homogenized, and Western blots were performed as already described [79,80,81]. The samples were stored at −80 °C for further analysis.

Specific primary antibodies against Aggrecan (sc-166951, Santa Cruz Biotechnology), NGF (MA5-32067, Thermo Fisher, Milan, Italy), trkA (JJ084-04, Thermo Fisher), WNT3a (sc-80457, Santa Cruz Biotechnology), anti-FZ8 (Bioworld Technology, St. Louis Park, MN, USA), anti–β-catenin (610153, BD Biosciences), anti-active β-catenin (05-665, Millipore, Milan, Italy), anti-NFkB (Santa Cruz Biotechnology, sc-8008), anti-NOS2 (Santa Cruz Biotechnology, sc-7271), or anti-COX-2 (Santa Cruz Biotechnology, sc-376861) were mixed in a 5% *w*/*v* nonfat dried milk solution and incubated with the membranes at 4 °C overnight. Afterwards, the blots were incubated with peroxidase-conjugated bovine anti-mouse IgG secondary antibodies or peroxidase-conjugated goat anti-rabbit IgG (Jackson Immuno Research, West Grove, PA, USA) for 1 h at room temperature [82]. The membranes were also incubated with antibodies against β-actin or lamin A/C (Santa Cruz Biotechnology) to verify that equal amounts of protein were loaded [83]. Images of the blot signals were imported into an analysis software (v2003, Image Quant TL, Milan, Italy) [84].

### 4.9. ELISA

The spinal cord levels of IL-18, TNF-α, IL-1β and PGE2 were measured by ELISA kits (R&D Systems), according to the manufacturer’s instructions [85].

### 4.10. Biochemical Analysis

Spinal cord tissues were weighed, homogenized, diluted to 10% (vol/vol) with a solution (Ca2+- and Mg2+-free PBS (pH 7.4)) and centrifuged. The supernatant was collected to detect biochemical indices [86]. SOD activity was evaluated using a xanthine oxidase solution and measured at 550 nm. The MDA level was quantified using modified thiobarbituric acid, and the absorbance was measured at 532 nm. The GSH content was evaluated using modified dithiobis(nitrobenzoic) acid, and the absorbance was measured at 420 nm. CAT activity was evaluated by a hydrogen peroxide solution, and the absorbance was measured at 240 nm.

### 4.11. Statistical Evaluation

All values in the figures and text are expressed as mean ± standard error of the mean (SEM) of N observations. For the in vivo studies, N represents the number of animals studied. The results displayed in the figures are representative of at minimum 3 experiments performed on diverse in vivo experimental days.

## 5. Conclusions

In summary, our findings displayed the impact of resveratrol on the progression and development of LBP. In particular, Wnt signaling, among other pathways in the spinal cord, was associated with the reduction of pain-related behavioral features in rats treated with resveratrol through the modulation of TNF-α and IL-18 levels and the related spinal inflammation and oxidative stress.

## Data Availability

The data presented in this study are available on request from the corresponding author.

## References

[1] Hartvigsen J., Hancock M.J., Kongsted A., Louw Q., Ferreira M.L., Genevay S., Hoy D., Karppinen J., Pransky G., Sieper J. (2018). What low back pain is and why we need to pay attention. Lancet.

[2] Buchbinder R., van Tulder M., Öberg B., Costa L.M., Woolf A., Schoene M., Croft P., Hartvigsen J., Cherkin D., Foster N.E. (2018). Low back pain: A call for action. Lancet.

[3] Jackson T., Thomas S., Stabile V., Han X., Shotwell M., McQueen K. (2015). Prevalence of chronic pain in low-income and middle-income countries: A systematic review and meta-analysis. Lancet.

[4] Maher C., Underwood M., Buchbinder R. (2017). Non-specific low back pain. Lancet.

[5] Chou R. (2014). In the clinic. Low back pain. Ann. Intern. Med..

[6] Kaye A.D., Jones M.R., Kaye A.M., Ripoll J.G., Galan V., Beakley B.D., Calixto F., Bolden J.L., Urman R.D., Manchikanti L. (2017). Prescription Opioid Abuse in Chronic Pain: An Updated Review of Opioid Abuse Predictors and Strategies to Curb Opioid Abuse: Part 1. Pain Physician.

[7] Han B., Compton W.M., Blanco C., Crane E., Lee J., Jones C.M. (2017). Prescription Opioid Use, Misuse, and Use Disorders in U.S. Adults: 2015 National Survey on Drug Use and Health. Ann. Intern. Med..

[8] Zhang Y.K., Huang Z.J., Liu S., Liu Y.P., Song A.A., Song X.J. (2013). WNT signaling underlies the pathogenesis of neuropathic pain in rodents. J. Clin. Investig..

[9] Ciani L., Salinas P.C. (2005). WNTs in the vertebrate nervous system: From patterning to neuronal connectivity. Nat. Rev. Neurosci..

[10] Packard M., Koo E.S., Gorczyca M., Sharpe J., Cumberledge S., Budnik V. (2002). The Drosophila Wnt, wingless, provides an essential signal for pre- and postsynaptic differentiation. Cell.

[11] Yang K., Wang X., Zhang H., Wang Z., Nan G., Li Y., Zhang F., Mohammed M.K., Haydon R.C., Luu H.H. (2016). The evolving roles of canonical WNT signaling in stem cells and tumorigenesis: Implications in targeted cancer therapies. Lab. Investig..

[12] Kikuchi A., Yamamoto H., Sato A., Matsumoto S. (2011). New insights into the mechanism of Wnt signaling pathway activation. Int. Rev. Cell Mol. Biol..

[13] Clevers H., Nusse R. (2012). Wnt/beta-catenin signaling and disease. Cell.

[14] Zhang Y., Zhao D., Li X., Gao B., Sun C., Zhou S., Ma Y., Chen X., Xu D. (2021). The Wnt/beta-Catenin Pathway Regulated Cytokines for Pathological Neuropathic Pain in Chronic Compression of Dorsal Root Ganglion Model. Neural. Plast..

[15] Xie H., Jing Y., Xia J., Wang X., You C., Yan J. (2016). Aquaporin 3 protects against lumbar intervertebral disc degeneration via the Wnt/beta-catenin pathway. Int. J. Mol. Med..

[16] Wagner R., Myers R.R. (1996). Endoneurial injection of TNF-alpha produces neuropathic pain behaviors. Neuroreport.

[17] Sommer C., Schmidt C., George A. (1998). Hyperalgesia in experimental neuropathy is dependent on the TNF receptor 1. Exp. Neurol..

[18] Ignatowski T.A., Covey W.C., Knight P.R., Severin C.M., Nickola T.J., Spengler R.N. (1999). Brain-derived TNFalpha mediates neuropathic pain. Brain Res..

[19] Ji R.R., Strichartz G. (2004). Cell signaling and the genesis of neuropathic pain. Sci. Stke.

[20] Watkins L.R., Maier S.F. (2005). Immune regulation of central nervous system functions: From sickness responses to pathological pain. J. Intern. Med..

[21] Zelenka M., Schafers M., Sommer C. (2005). Intraneural injection of interleukin-1beta and tumor necrosis factor-alpha into rat sciatic nerve at physiological doses induces signs of neuropathic pain. Pain.

[22] Roberts R.A., Smith R.A., Safe S., Szabo C., Tjalkens R.B., Robertson F.M. (2010). Toxicological and pathophysiological roles of reactive oxygen and nitrogen species. Toxicology.

[23] Zhao Y., Song W., Wang Z., Wang Z., Jin X., Xu J., Bai L., Li Y., Cui J., Cai L. (2018). Resveratrol attenuates testicular apoptosis in type 1 diabetic mice: Role of Akt-mediated Nrf2 activation and p62-dependent Keap1 degradation. Redox Biol..

[24] Orlandi I., Stamerra G., Strippoli M., Vai M. (2017). During yeast chronological aging resveratrol supplementation results in a short-lived phenotype Sir2-dependent. Redox Biol..

[25] Zhu Y., Feng B., He S., Su Z., Zheng G. (2018). Resveratrol combined with total flavones of hawthorn alleviate the endothelial cells injury after coronary bypass graft surgery. Phytomedicine.

[26] Tsai C.C., Tey S.L., Lee M.C., Liu C.W., Su Y.T., Huang S.C. (2018). Mechanism of resveratrol-induced relaxation of the guinea pig fundus. Phytomedicine.

[27] Jeong J.B., Lee J., Lee S.H. (2015). TCF4 Is a Molecular Target of Resveratrol in the Prevention of Colorectal Cancer. Int. J. Mol. Sci..

[28] Hope C., Planutis K., Planutiene M., Moyer M.P., Johal K.S., Woo J., Santoso C., Hanson J.A., Holcombe R.F. (2008). Low concentrations of resveratrol inhibit Wnt signal throughput in colon-derived cells: Implications for colon cancer prevention. Mol. Nutr. Food Res..

[29] Cilibrasi C., Riva G., Romano G., Cadamuro M., Bazzoni R., Butta V., Paoletta L., Dalpra L., Strazzabosco M., Lavitrano M. (2017). Resveratrol Impairs Glioma Stem Cells Proliferation and Motility by Modulating the Wnt Signaling Pathway. PLoS ONE.

[30] Yang H.C., Wang J.Y., Bu X.Y., Yang B., Wang B.Q., Hu S., Yan Z.Y., Gao Y.S., Han S.Y., Qu M.Q. (2019). Resveratrol restores sensitivity of glioma cells to temozolamide through inhibiting the activation of Wnt signaling pathway. J. Cell Physiol..

[31] Liu Z.L., Li H., Liu J., Wu M.L., Chen X.Y., Liu L.H., Wang Q. (2017). Inactivated Wnt signaling in resveratrol-treated epidermal squamous cancer cells and its biological implication. Oncol. Lett..

[32] Xie D., Zheng G.Z., Xie P., Zhang Q.H., Lin F.X., Chang B., Hu Q.X., Du S.X., Li X.D. (2017). Antitumor activity of resveratrol against human osteosarcoma cells: A key role of Cx43 and Wnt/beta-catenin signaling pathway. Oncotarget.

[33] Liu H., Zhang S., Zhao L., Zhang Y., Li Q., Chai X., Zhang Y. (2016). Resveratrol Enhances Cardiomyocyte Differentiation of Human Induced Pluripotent Stem Cells through Inhibiting Canonical WNT Signal Pathway and Enhancing Serum Response Factor-miR-1 Axis. Stem. Cells Int..

[34] Oz B., Yildirim A., Yolbas S., Celik Z.B., Etem E.O., Deniz G., Akin M., Akar Z.A., Karatas A., Koca S.S. (2019). Resveratrol inhibits Src tyrosine kinase, STAT3, and Wnt signaling pathway in collagen induced arthritis model. Biofactors.

[35] Bo S., Ciccone G., Castiglione A., Gambino R., De Michieli F., Villois P., Durazzo M., Cavallo-Perin P., Cassader M. (2013). Anti-inflammatory and antioxidant effects of resveratrol in healthy smokers a randomized, double-blind, placebo-controlled, cross-over trial. Curr. Med. Chem..

[36] Gerszon J., Rodacka A., Puchała M. (2014). Antioxidant properties of resveratrol and its protective effects in neurodegenerative diseases. Adv. Cell Biol..

[37] Banez M.J., Geluz M.I., Chandra A., Hamdan T., Biswas O.S., Bryan N.S., Von Schwarz E.R. (2020). A systemic review on the antioxidant and anti-inflammatory effects of resveratrol, curcumin, and dietary nitric oxide supplementation on human cardiovascular health. Nutr. Res..

[38] Park M., Shen K. (2012). WNTs in synapse formation and neuronal circuitry. Embo. J..

[39] Itokazu T., Hayano Y., Takahashi R., Yamashita T. (2014). Involvement of Wnt/beta-catenin signaling in the development of neuropathic pain. Neurosci. Res..

[40] Degenhardt B.F., Johnson J.C., Fossum C., Andicochea C.T., Stuart M.K. (2017). Changes in Cytokines, Sensory Tests, and Self-reported Pain Levels After Manual Treatment of Low Back Pain. Clin. Spine. Surg..

[41] Weber K.T., Satoh S., Alipui D.O., Virojanapa J., Levine M., Sison C., Quraishi S., Bloom O., Chahine N.O. (2015). Exploratory study for identifying systemic biomarkers that correlate with pain response in patients with intervertebral disc disorders. Immunol. Res..

[42] Koch A., Zacharowski K., Boehm O., Stevens M., Lipfert P., von Giesen H.J., Wolf A., Freynhagen R. (2007). Nitric oxide and pro-inflammatory cytokines correlate with pain intensity in chronic pain patients. Inflamm. Res..

[43] Lundin E., Dossus L., Clendenen T., Krogh V., Grankvist K., Wulff M., Sieri S., Arslan A.A., Lenner P., Berrino F. (2009). C-reactive protein and ovarian cancer: A prospective study nested in three cohorts (Sweden, USA, Italy). Cancer Causes Control..

[44] Uceyler N., Eberle T., Rolke R., Birklein F., Sommer C. (2007). Differential expression patterns of cytokines in complex regional pain syndrome. Pain.

[45] Uceyler N., Rogausch J.P., Toyka K.V., Sommer C. (2007). Differential expression of cytokines in painful and painless neuropathies. Neurology.

[46] Merlin J.S., Westfall A.O., Heath S.L., Goodin B.R., Stewart J.C., Sorge R.E., Younger J. (2017). Brief Report: IL-1beta Levels Are Associated With Chronic Multisite Pain in People Living With HIV. J. Acquir. Immune. Defic. Syndr..

[47] Rannou F., Ouanes W., Boutron I., Lovisi B., Fayad F., Mace Y., Borderie D., Guerini H., Poiraudeau S., Revel M. (2007). High-sensitivity C-reactive protein in chronic low back pain with vertebral end-plate Modic signal changes. Arthritis Rheum.

[48] Sandireddy R., Yerra V.G., Areti A., Komirishetty P., Kumar A. (2014). Neuroinflammation and oxidative stress in diabetic neuropathy: Futuristic strategies based on these targets. Int. J. Endocrinol..

[49] Schinkel C., Scherens A., Koller M., Roellecke G., Muhr G., Maier C. (2009). Systemic inflammatory mediators in post-traumatic complex regional pain syndrome (CRPS I)—longitudinal investigations and differences to control groups. Eur. J. Med. Res..

[50] Sibille K.T., Steingrimsdottir O.A., Fillingim R.B., Stubhaug A., Schirmer H., Chen H., McEwen B.S., Nielsen C.S. (2016). Investigating the Burden of Chronic Pain: An Inflammatory and Metabolic Composite. Pain Res. Manag..

[51] Messier S.P., Mihalko S.L., Legault C., Miller G.D., Nicklas B.J., DeVita P., Beavers D.P., Hunter D.J., Lyles M.F., Eckstein F. (2013). Effects of intensive diet and exercise on knee joint loads, inflammation, and clinical outcomes among overweight and obese adults with knee osteoarthritis: The IDEA randomized clinical trial. JAMA.

[52] Richard C., Couture P., Desroches S., Lamarche B. (2013). Effect of the Mediterranean diet with and without weight loss on markers of inflammation in men with metabolic syndrome. Obesity.

[53] Allison D.J., Thomas A., Beaudry K., Ditor D.S. (2016). Targeting inflammation as a treatment modality for neuropathic pain in spinal cord injury: A randomized clinical trial. J. Neuroinflamm..

[54] Goodin B.R., Quinn N.B., Kronfli T., King C.D., Page G.G., Haythornthwaite J.A., Edwards R.R., Stapleton L.M., McGuire L. (2012). Experimental pain ratings and reactivity of cortisol and soluble tumor necrosis factor-alpha receptor II following a trial of hypnosis: Results of a randomized controlled pilot study. Pain Med..

[55] Lee K.M., Kang B.S., Lee H.L., Son S.J., Hwang S.H., Kim D.S., Park J.S., Cho H.J. (2004). Spinal NF-kB activation induces COX-2 upregulation and contributes to inflammatory pain hypersensitivity. Eur. J. Neurosci..

[56] Dolan S., Field L.C., Nolan A.M. (2000). The role of nitric oxide and prostaglandin signaling pathways in spinal nociceptive processing in chronic inflammation. Pain.

[57] Gregersen S., Samocha-Bonet D., Heilbronn L.K., Campbell L.V. (2012). Inflammatory and oxidative stress responses to high-carbohydrate and high-fat meals in healthy humans. J. Nutr. Metab..

[58] Levitan E.B., Cook N.R., Stampfer M.J., Ridker P.M., Rexrode K.M., Buring J.E., Manson J.E., Liu S. (2008). Dietary glycemic index, dietary glycemic load, blood lipids, and C-reactive protein. Metabolism.

[59] Nasto L.A., Robinson A.R., Ngo K., Clauson C.L., Dong Q., St Croix C., Sowa G., Pola E., Robbins P.D., Kang J. (2013). Mitochondrial-derived reactive oxygen species (ROS) play a causal role in aging-related intervertebral disc degeneration. J. Orthop. Res..

[60] Kaushik A.S., Strath L.J., Sorge R.E. (2020). Dietary Interventions for Treatment of Chronic Pain: Oxidative Stress and Inflammation. Pain.

[61] Bresciani G., da Cruz I.B., Gonzalez-Gallego J. (2015). Manganese superoxide dismutase and oxidative stress modulation. Adv. Clin. Chem..

[62] Jana T., Tzveta S., Zlatina N., Natasha I., Dimitrinka A., Milena A., Katerina G. (2020). Effect of endurance training on diurnal rhythms of superoxide dismutase activity, glutathione and lipid peroxidation in plasma of pinealectomized rats. Neurosci. Lett..

[63] Lee M., Kim B.J., Lim E.J., Back S.K., Lee J.H., Yu S.W., Hong S.H., Kim J.H., Lee S.H., Jung W.W. (2009). Complete Freund’s adjuvant-induced intervertebral discitis as an animal model for discogenic low back pain. Anesth. Analg..

[64] Wang G., Hu Z., Song X., Cui Q., Fu Q., Jia R., Zou Y., Li L., Yin Z. (2017). Analgesic and Anti-Inflammatory Activities of Resveratrol through Classic Models in Mice and Rats. Evid. Based Complement. Altern. Med..

[65] Fusco R., Siracusa R., D’Amico R., Peritore A.F., Cordaro M., Gugliandolo E., Crupi R., Impellizzeri D., Cuzzocrea S., Di Paola R. (2019). Melatonin Plus Folic Acid Treatment Ameliorates Reserpine-Induced Fibromyalgia: An Evaluation of Pain, Oxidative Stress, and Inflammation. Antioxidants.

[66] Cordaro M., Siracusa R., Impellizzeri D., D’Amico R., Peritore A.F., Crupi R., Gugliandolo E., Fusco R., Di Paola R., Schievano C. (2019). Safety and efficacy of a new micronized formulation of the ALIAmide palmitoylglucosamine in preclinical models of inflammation and osteoarthritis pain. Arthritis Res..

[67] Fusco R., Siracusa R., Peritore A.F., Gugliandolo E., Genovese T., D’Amico R., Cordaro M., Crupi R., Mandalari G., Impellizzeri D. (2020). The Role of Cashew (*Anacardium occidentale* L.) Nuts on an Experimental Model of Painful Degenerative Joint Disease. Antioxidants.

[68] Gugliandolo E., D’Amico R., Cordaro M., Fusco R., Siracusa R., Crupi R., Impellizzeri D., Cuzzocrea S., Di Paola R. (2018). Effect of PEA-OXA on neuropathic pain and functional recovery after sciatic nerve crush. J. Neuroinflamm..

[69] Britti D., Crupi R., Impellizzeri D., Gugliandolo E., Fusco R., Schievano C., Morittu V.M., Evangelista M., Di Paola R., Cuzzocrea S. (2017). A novel composite formulation of palmitoylethanolamide and quercetin decreases inflammation and relieves pain in inflammatory and osteoarthritic pain models. BMC Vet. Res..

[70] Impellizzeri D., Peritore A.F., Cordaro M., Gugliandolo E., Siracusa R., Crupi R., D’Amico R., Fusco R., Evangelista M., Cuzzocrea S. (2019). The neuroprotective effects of micronized PEA (PEA-m) formulation on diabetic peripheral neuropathy in mice. FASEB J..

[71] Fusco R., Gugliandolo E., Campolo M., Evangelista M., Di Paola R., Cuzzocrea S. (2017). Effect of a new formulation of micronized and ultramicronized N-palmitoylethanolamine in a tibia fracture mouse model of complex regional pain syndrome. PLoS ONE.

[72] Masuda K., Aota Y., Muehleman C., Imai Y., Okuma M., Thonar E.J., Andersson G.B., An H.S. (2005). A novel rabbit model of mild, reproducible disc degeneration by an anulus needle puncture: Correlation between the degree of disc injury and radiological and histological appearances of disc degeneration. Spine.

[73] Whiting P.F., Wolff R.F., Deshpande S., Di Nisio M., Duffy S., Hernandez A.V., Keurentjes J.C., Lang S., Misso K., Ryder S. (2015). Cannabinoids for Medical Use: A Systematic Review and Meta-analysis. JAMA.

[74] Peritore A.F., Siracusa R., Fusco R., Gugliandolo E., D’Amico R., Cordaro M., Crupi R., Genovese T., Impellizzeri D., Cuzzocrea S. (2020). Ultramicronized Palmitoylethanolamide and Paracetamol, a New Association to Relieve Hyperalgesia and Pain in a Sciatic Nerve Injury Model in Rat. Int. J. Mol. Sci..

[75] D’Amico R., Fusco R., Cordaro M., Siracusa R., Peritore A.F., Gugliandolo E., Crupi R., Scuto M., Cuzzocrea S., Di Paola R. (2020). Modulation of NLRP3 Inflammasome through Formyl Peptide Receptor 1 (Fpr-1) Pathway as a New Therapeutic Target in Bronchiolitis Obliterans Syndrome. Int. J. Mol. Sci..

[76] Fusco R., Gugliandolo E., Siracusa R., Scuto M., Cordaro M., D’Amico R., Evangelista M., Peli A., Peritore A.F., Impellizzeri D. (2020). Formyl Peptide Receptor 1 Signaling in Acute Inflammation and Neural Differentiation Induced by Traumatic Brain Injury. Biology.

[77] Fusco R., Cordaro M., Siracusa R., D’Amico R., Genovese T., Gugliandolo E., Peritore A.F., Crupi R., Impellizzeri D., Cuzzocrea S. (2020). Biochemical Evaluation of the Antioxidant Effects of Hydroxytyrosol on Pancreatitis-Associated Gut Injury. Antioxidants.

[78] Gugliandolo E., Fusco R., D’Amico R., Militi A., Oteri G., Wallace J.L., Di Paola R., Cuzzocrea S. (2018). Anti-inflammatory effect of ATB-352, a H2S -releasing ketoprofen derivative, on lipopolysaccharide-induced periodontitis in rats. Pharm. Res..

[79] Di Paola R., Impellizzeri D., Fusco R., Cordaro M., Siracusa R., Crupi R., Esposito E., Cuzzocrea S. (2016). Ultramicronized palmitoylethanolamide (PEA-um((R))) in the treatment of idiopathic pulmonary fibrosis. Pharm. Res..

[80] Siracusa R., Fusco R., Peritore A.F., Cordaro M., D’Amico R., Genovese T., Gugliandolo E., Crupi R., Smeriglio A., Mandalari G. (2020). The Antioxidant and Anti-Inflammatory Properties of Anacardium occidentale L. Cashew Nuts in a Mouse Model of Colitis. Nutrients.

[81] Fusco R., Cordaro M., Genovese T., Impellizzeri D., Siracusa R., Gugliandolo E., Peritore A.F., D’Amico R., Crupi R., Cuzzocrea S. (2020). Adelmidrol: A New Promising Antioxidant and Anti-Inflammatory Therapeutic Tool in Pulmonary Fibrosis. Antioxidants.

[82] Gugliandolo E., Fusco R., Ginestra G., D’Amico R., Bisignano C., Mandalari G., Cuzzocrea S., Di Paola R. (2019). Involvement of TLR4 and PPAR-alpha Receptors in Host Response and NLRP3 Inflammasome Activation, Against Pulmonary Infection With Pseudomonas Aeruginosa. Shock.

[83] Gugliandolo E., Fusco R., D’Amico R., Peditto M., Oteri G., Di Paola R., Cuzzocrea S., Navarra M. (2018). Treatment With a Flavonoid-Rich Fraction of Bergamot Juice Improved Lipopolysaccharide-Induced Periodontitis in Rats. Front. Pharm..

[84] Fusco R., Gugliandolo E., Biundo F., Campolo M., Di Paola R., Cuzzocrea S. (2017). Inhibition of inflammasome activation improves lung acute injury induced by carrageenan in a mouse model of pleurisy. FASEB J..

[85] D’Amico R., Fusco R., Siracusa R., Impellizzeri D., Peritore A.F., Gugliandolo E., Interdonato L., Sforza A.M., Crupi R., Cuzzocrea S. (2021). Inhibition of P2X7 Purinergic Receptor Ameliorates Fibromyalgia Syndrome by Suppressing NLRP3 Pathway. Int. J. Mol. Sci..

[86] Zhou K.L., Chen D.H., Jin H.M., Wu K., Wang X.Y., Xu H.Z., Zhang X.L. (2016). Effects of calcitriol on experimental spinal cord injury in rats. Spinal Cord.

